# Polyprotein Processing as a Determinant for In Vitro Activity of Semliki Forest Virus Replicase

**DOI:** 10.3390/v9100292

**Published:** 2017-10-07

**Authors:** Maija K. Pietilä, Irina C. Albulescu, Martijn J. van Hemert, Tero Ahola

**Affiliations:** 1 Department of Food and Environmental Sciences, University of Helsinki, Viikinkaari 9 P.O. Box 56, 00014 Helsinki, Finland; maija.pietila@helsinki.fi; 2 Department of Medical Microbiology, Leiden University Medical Center P.O. Box 9600, 2300 RC, Leiden, The Netherlands; i.c.albulescu@lumc.nl

**Keywords:** alphavirus, Semliki Forest virus, nonstructural protein, polymerase, replication complex, in vitro replication, RNA synthesis

## Abstract

Semliki Forest virus (SFV) is an arthropod-borne alphavirus that induces membrane invaginations (spherules) in host cells. These harbor the viral replication complexes (RC) that synthesize viral RNA. Alphaviruses have four replicase or nonstructural proteins (nsPs), nsP1–4, expressed as polyprotein P1234. An early RC, which synthesizes minus-strand RNA, is formed by the polyprotein P123 and the polymerase nsP4. Further proteolytic cleavage results in a late RC consisting of nsP1–4 and synthesizing plus strands. Here, we show that only the late RCs are highly active in RNA synthesis in vitro. Furthermore, we demonstrate that active RCs can be isolated from both virus-infected cells and cells transfected with the wild-type replicase in combination with a plasmid expressing a template RNA. When an uncleavable polyprotein P123 and polymerase nsP4 were expressed together with a template, high levels of minus-strand RNA were produced in cells, but RCs isolated from these cells were hardly active in vitro. Furthermore, we observed that the uncleavable polyprotein P123 and polymerase nsP4, which have previously been shown to form spherules even in the absence of the template, did not replicate an exogenous template. Consequently, we hypothesize that the replicase proteins were sequestered in spherules and were no longer able to recruit a template.

## 1. Introduction

Positive-strand RNA (+RNA) viruses are the most common type of RNA viruses, and include numerous human pathogens [1]. The recent re-emergence and outbreaks of chikungunya virus (CHIKV) highlight the medical significance of alphaviruses, which belong to the alphavirus-like superfamily containing several families of +RNA viruses from animals or plants [2,3,4]. Alphaviruses form the family *Togaviridae* together with the rubella virus, and they are mainly arthropod-borne viruses that infect vertebrates. The alphavirus genome of about 11–12 kb contains a 5′ cap and encodes two polyproteins. The P1234 polyprotein is processed into four nonstructural proteins (nsPs), nsP1–4, and the structural polyprotein, translated from a subgenomic RNA, is cleaved to yield the capsid and envelope proteins [5]. All four nsPs are required for viral RNA synthesis [6,7,8], and they form a membrane-associated replication complex (RC). nsP1 is the RNA-capping enzyme and the only viral protein responsible for anchoring the RC to membranes; nsP2 is the helicase and protease; nsP3 interacts with several host factors; and nsP4 is the core RNA-dependent RNA polymerase [9,10,11,12,13,14,15,16]. All members of the alphavirus-like superfamily share capping, helicase, and polymerase domains in their replicase proteins [2].

Membrane-associated RCs are typical of all eukaryotic +RNA viruses. Membrane connection is indispensable, and two types of replication-associated membrane modifications have been recognized: membrane invaginations called spherules, as e.g., induced by alphaviruses; and double-membrane vesicles (DMVs), as e.g., induced by coronaviruses [17]. Alphaviruses form spherules at the plasma membrane, and CHIKV and Sindbis virus (SINV) spherules predominantly stay at the plasma membrane, while Semliki Forest virus (SFV) spherules are internalized to endo- and lysosomal membranes [18,19,20].

The early RCs of alphaviruses are formed when the polymerase nsP4 is first cleaved from the nonstructural polyprotein. The resulting complex of P123 and nsP4 mainly synthesizes minus-strand RNA using the genomic RNA as a template [8,21]. Next, P123 is cleaved to individual proteins, and the fully processed nsPs form the late RC, which synthesizes only genomic and subgenomic plus strands from the minus-strand RNA [8]. It has been shown that the template’s length determines SFV spherule size [22], and the current view is that the minus-strand RNA synthesized from the genomic template is inside the mature spherule as part of double-stranded RNA (dsRNA) molecules that form the replicative intermediates, and newly made plus strands are released into the cytoplasm [23,24,25].

Plasmid-based trans-replication systems have been developed to study the formation and activity of alphaviral RCs and the functions of the nsPs outside the context of viral infection [19,22,24,26,27,28,29]. The replicase proteins are expressed from one or several plasmids together with a separate plasmid that expresses a template RNA that can be replicated in trans. However, active replication is achieved only when plus-stranded RNA is expressed as a template. This indicates that replication cannot initiate from a single-stranded minus strand as a template, but rather requires a dsRNA intermediate for plus-strand synthesis [27,30]. Furthermore, it has been recently shown that a partially uncleaved alphavirus replicase can form spherules in the absence of the RNA template [29]. The template-independent spherule formation requires a combination of the uncleaved P123 and nsP4 or nsP1, or uncleaved P23 and nsP4. Furthermore, even a polymerase mutant (nsP4^GAA^) with its active site Gly-Asp-Asp (GDD) mutated to Gly-Ala-Ala (GAA) causes spherule formation when co-expressed with P123 or nsP1 and P23. Spherule morphology in the absence of the template is, however, more irregular compared to those formed during normal replication, confirming the idea that the template plays a central role in size determination.

The RNA-synthesizing activity of alphavirus RCs has also been studied using in vitro replication assays (IVRAs). CHIKV, SINV, and SFV synthesize single-stranded genomic and subgenomic RNA as well as double-stranded RNA in vitro [25,31,32], and for CHIKV it has been shown that in vitro any produced RNA is of positive polarity [25]. Besides genomic and subgenomic RNA, CHIKV and other alphaviruses synthesize an RNA species of about 7.5 kb [25,33]. This RNA II contains the 5′ end of the genome up to the subgenomic promoter. Although RNA II is produced both in cells and in vitro, its function remains an unanswered question [25].

Here, we applied the IVRA developed for CHIKV [25] to study the RNA synthesis of SFV in the trans-replication system. With the assay, robust activity could be measured in samples prepared from SFV-infected cells. The subsequent in vitro characterization of RCs formed upon the expression of different SFV replicase constructs using the trans-replication system revealed that only the fully processed polyprotein supports highly active RNA synthesis.

## 2. Materials and Methods

### 2.1. Cell Culture, Plasmids, and Viruses

BHK (baby hamster kidney)-21 cells were cultured in Dulbecco’s modified Eagle’s medium (DMEM; Sigma-Aldrich, St. Louis, MS, USA) supplemented with 10% (*v*/*v*) fetal bovine serum (FBS; Gibco, Baltimore, MD, USA), 2 mM l-glutamine (Gibco), 100 U/mL penicillin (Gibco), and 100 µg/mL streptomycin (Gibco). BSR T7/5 cells, a derivative of BHK-21 cells stably expressing T7 RNA polymerase [34], were maintained in DMEM supplemented with 10% FBS, 2% (*v*/*v*) Difco tryptose phosphate broth (Becton, Dickinson and Company, Franklin Lakes, NJ, USA), 2 mM l-glutamine, 1% (*v*/*v*) non-essential amino acids (Gibco), 1 mg/mL G418 (Merck, Kenilworth, NJ, USA), 100 U/mL penicillin, and 100 μg/mL streptomycin (Gibco). Both cell lines were grown at 37 °C and in 5% CO_2_. Virus-infected or transfected cells were grown in the same conditions.

All virus and plasmid constructs used in this study contain a hemagglutinin (HA) peptide (YPYDVPDYA) in a frame within nsP3. For generation of SFV4-HA virus, the oligos encoding the HA-tag and flanking glycines were 5′-TCGAGGGCTACCCATACGATGTTCCAGATTACGCTGGTG-3′ and 5′-TCGACACCAGCGTAATCTGGAACATCGTATGGGTAGCCC-3′. Oligos were annealed, phosphorylated, and cloned into the XhoI site of replicon pSFV1-ZsG, removing the ZsGreen gene [18]. A larger piece containing the HA-insert was then transferred with SacI and NotI to pCMV-SFV4 [35]. The virus stocks were produced by transfecting ~3 × 10^6^ BHK-21 cells with 2.5 µg of pCMV-SFV4-HA using Lipofectamine LTX (Invitrogen, Carlsbad, CA, USA) according to the manufacturer’s instructions. After 16–18 h incubation in minimum essential medium (MEM; Gibco) containing 0.2% (*v*/*v*) bovine serum albumin (BSA) and 2 mM l-glutamine, the supernatant was collected. This primary stock was used to infect BHK-21 cells at a multiplicity of infection (MOI) of 0.01 in MEM containing 0.2% BSA and 2 mM l-glutamine. After 1 h, cells were washed twice and new medium was added. The supernatant designated as secondary stock was collected after 16–18 h post infection (p.i.). Titers were quantified by plaque assay titration [36].

To generate HA-tagged replicase polyprotein constructs, the ZsGreen gene in constructs P123Z4 and P1^2^3Z4 [26] was replaced with an HA tag-containing Bsu36I-AgeI restriction fragment from the replicon. In order to obtain a P123^HA^4^GAA^ expression construct, a NotI-BamHI fragment of nsP4 containing GAA substitution was transferred from P1234^GAA^ [26] to P123^HA^4. For the construction of P12^CA^3^HA^, Bsu36I and AgeI were used to replace ZsGreen in P12^CA^3Z [24] with the HA-tag. The ubi-nsP4 and the template Tmed constructs have been described in [26].

### 2.2. Metabolic Labeling with ^3^H-Uridine

BHK-21 cells were infected using the secondary stock of SFV4-HA at an MOI of 50 for 1 h in PBS containing 2% (*v*/*v*) fetal calf serum (FCS; Bodinco, Alkmaar, The Netherlands). After the adsorption, cells were washed and DMEM containing 2% FCS was added. Mock-infected cells served as a control. Thirty minutes before labeling, cellular transcription was inhibited by adding Actinomycin D (5 µg/mL; Sigma-Aldrich). Metabolic pulse-labeling of approximately 6 × 10^5^ mock- or SFV-infected cells was performed by incubating cells with 40 µCi of ^3^H-uridine (Perkin Elmer, Ayer Rajah, Singapore) in medium for 1 h. After the labeling, cells were lysed in LiDS/LET (5% lithium dodecyl sulfate, 20 mM Tris-HCl (pH 7.4), 100 mM LiCl, 2 mM EDTA, and 5 mM DTT) and treated with proteinase K (80 µg/mL; Thermo Fisher Scientific, Waltham, MA, USA) for 15 min at 42 °C. Total RNA was isolated using the acid phenol method and analysed by denaturing agarose gel electrophoresis as described below. Fluorographic detection of ^3^H-labeled RNA was performed as described in [37]. Incorporation of ^3^H-uridine was also quantified from 2-µL samples of isolated total RNA with a liquid scintillation counter (Beckman LS 6500 IC, Brea, CA, USA).

### 2.3. Isolation of RCs from Infected or Transfected Cells

For virus infection, the secondary stock of SFV4-HA was used to infect approximately 1 × 10^7^ BHK-21 cells at an MOI of 50 for 1 h in MEM containing 0.2% BSA and 2 mM l-glutamine. After the adsorption, cells were washed twice and new medium was added. Mock-infected cells served as a control. Cells were harvested by trypsinization at 4 h p.i., washed twice with phosphate-buffered saline, and resuspended in 0.5 mL of dilution buffer (DB) (35 mM HEPES (pH 7.4), 250 mM sucrose, 2.5 mM DTT, 7 mM KCl) containing 2 μg/mL Actinomycin D, 200 U/mL RiboLock (Thermo Fisher Scientific), and Pierce EDTA-Free Protease Inhibitor (1 tablet per 10 mL; Thermo Fisher Scientific). After a 20-min incubation on ice, cells were disrupted using a Dounce homogenizer with 40 strokes and unlysed cells and nuclei were removed by centrifugation (510× *g*, 10 min, 4 °C) yielding a post-nuclear supernatant (PNS). A membrane pellet (P15) and cytosolic fraction (S15) were prepared by further centrifugation of PNS (15,000× *g*, 10 min, 4 °C). The P15 fraction was washed once with DB containing Actinomycin D, RiboLock, and protease inhibitor and resuspended in the same buffer.

For the trans-replication system, BSR cells were co-transfected with the replicase and Tmed template plasmids containing the T7 promoter [26]. LTX was used according to the manufacturer’s instructions. Per 10-cm dish containing approximately 4 × 10^6^ cells, 5.5 µg of the replicase, and 6.9 µg of the template plasmid were co-transfected. Mock-transfected cells without LTX were used as a control. After a 16-h incubation in MEM containing 0.2% BSA and 2 mM l-glutamine, cells were harvested by trypsinization. PNS, P15, and S15 were prepared as described for virus infection.

### 2.4. Purification of Membranes

BSR cells were transfected with 12.4 µg of P1^2^3^HA^4 or P123^HA^4 plasmid DNA per 10-cm dish, cells were harvested at 6 or 16 h post transfection, and PNS was prepared as described above. HA-affinity capture was performed as described in [38], except that the elution was done by boiling the beads in Laemmli sample buffer. Membranes present in PNS samples were purified by flotation centrifugation (90,000× *g*, 18 h, 4 °C) in a discontinuous iodixanol (Sigma-Aldrich) gradient consisting of 1 mL DB, 9 mL 30% (*w*/*v*) iodixanol in DB, and 2 mL 35% iodixanol in DB. The final layer contained 0.5 mL PNS. Protease inhibitor was used throughout the purification. After the centrifugation, the light-scattering zone between DB and 30% iodixanol layers was collected and used for the Western blotting and replication assay described below.

### 2.5. IVRA, RNA Isolation, Agarose Gel Electrophoresis, and in-Gel Hybridization

IVRA was designed based on that described in [25]. Typically, 2-fold and 30-fold dilutions of PNS, P15, and S15 prepared from transfected and virus-infected cells were used, respectively. A standard 30-µL reaction contained 22 µL of PNS, P15, or S15, 1 mg/mL BSA, 28 mM HEPES (pH 7.4), 200 mM sucrose, 2 mM DTT, 5.6 mM KCl, 3 mM magnesium acetate, 1.5 μg/mL Actinomycin D, 810 U/mL RiboLock, 17 mM creatine phosphate (Sigma-Aldrich), 8.3 U/mL creatine phosphokinase (Sigma-Aldrich), 1 mM ATP, 10 µM UTP, 10 µM GTP, 8.5 µM CTP, and 0.055 µM (5 µCi) of α-^32^P-CTP (Perkin Elmer). For IVRA with exogenous template RNA, an in vitro transcript of Tmed was prepared using a SacI-linearized template plasmid and a mMESSAGE mMACHINE^®^ T7 Transcription Kit (Ambion, Thermo Fisher Scientific, Waltham, MA, USA) according to the manufacturer’s instructions, and 1 µg of RNA was added per reaction. After 1-h incubation at 30 °C, IVRA reactions were terminated by the addition of LiDS/LET containing 80 µg/mL proteinase K and incubating for 15 min at 37 °C. Unicorporated label was removed using RNase-free Micro Bio-Spin™ P-30 Gel Columns (Bio-rad, Berkeley, CA, USA), and RNA was isolated using the acid phenol method.

The acid phenol method was used to isolate total RNA from IVRA samples as well as cell lysates, PNS, P15, and S15 prepared from SFV-infected BHK-21 cells. RNA was extracted with acid phenol:chloroform, premixed with isoamyl alcohol (125:24:1 phenol:chloroform:isoamyl alcohol; pH 4.5; Ambion), precipitated with isopropyl alcohol, and washed with 70% ethanol. TRIzol^®^ Reagent (Invitrogen) was used to isolate total RNA from PNS, P15, and S15 fractions prepared from transfected BSR cells according to the manufacturer’s instructions. In both methods, GlycoBlue (Ambion) was used as a coprecipitant and isolated RNA was dissolved in 1 mM sodium citrate (pH 6.4; Ambion). Isolated RNA was analysed by denaturing formaldehyde agarose gel electrophoresis as described previously [37], except that 1.0% (*w*/*v*) agarose gels were used.

PNS, P15, and S15 fractions prepared from BSR cells co-transfected with P1^2^3^HA^4 and Tmed were treated with RNase Cocktail™ Enzyme Mix (Ambion) containing RNase A and T1 (final concentrations of 5 and 205 units/mL, respectively). Incubation was in DB (low salt conditions) for 15 min at 37 °C, and RNA was isolated using TRIzol^®^ Reagent and analysed as above.

In-gel hybridization to detect RNA molecules from dried gels with ^32^P-labeled oligonucleotides was performed as described in [37]. All hybridizations were done at 55 °C. Negative-stranded SFV4-HA RNA was detected with the probe HybSFV4neg_4 (5′-GTATCATGTGAAGGGTACGTAGTTAAGA-3′) that corresponds to nucleotides 926–953 of genomic RNA and thus recognizes the minus-strand RNA. Genomic and subgenomic RNA of SFV4-HA were detected with the probe HybSFV4pos_5 (5′-GCTTTGTTACAAGTTATACAGTTATGGTTATATG-3′) that is complementary to nucleotides 11,292–11,325 and thus detects the plus-strand RNA. Negative-stranded Tmed RNA was visualized using the probe HybSFV4neg (5′-CAAAAGATTTTGTTCCAGCTCCTG-3′) corresponding to nucleotides 28–51 of the Tmed transcript. Positive-stranded genomic and subgenomic RNA of Tmed were detected with the probe HybTmedpos_2 (5′-TTAAGCTCTAATTAATTAGTCGACCTACTTGTAC-3′) that is complementary to nucleotides 2807–2840. All 18S ribosomal RNA (rRNA) was detected with the oligonucleotide probe 5′-ATGCCCCCGGCCGTCCCTCT-3′. If the same gel was hybridized with different probes, the gel was stripped between hybridizations by incubating in 1% SDS at 80 °C and confirming that no signal remained by phosphorimaging as described below.

All ^32^P-labeled RNA was detected by exposing Phosphor Imager screens to dried gels and scanning the screens with a Typhoon 9410 imager (GE Healthcare, Chicago, IL, USA). Incorporated label was quantified by Quantity One software (Bio-rad).

### 2.6. Hybridization of ^32^P-labeled RNA Products with Capture Probes

In vitro transcripts containing nucleotides 8390–11,468 of SFV4-HA genomic RNA (capture probe for minus strands) and its complementary sequence (capture probe for plus strands), nucleotides 1–2963 of Tmed RNA (capture probe for minus strands), nucleotides 1–2878 of Tmed negative-sense RNA (capture probe for plus strands), and nucleotides 1–2042 of equine arteritis virus (EAV) genome were prepared using mMESSAGE mMACHINE^®^ T7 or SP6 Transcription Kit (Ambion) according to the manufacturer’s instructions. SacI-linearized Tmed or XhoI-linearized pEAV221Δ plasmids were used as templates for Tmed and EAV in vitro plus transcripts, respectively. pEAV221Δ was obtained by cleaving pEAV221 [39] with HindIII and ligating the 4729-bp fragment. For Tmed minus transcript, a PCR product made using oligos 5′-ATGTGGCAACATTTAGGTGACACTATAGAAGAATTGCAAAATAAAATAAAAATC-3′ and 5′-ATGACGGATGTGTGACATAC-3′ was used as a template. For SFV4-HA, a PCR product made using oligos 5′-ACGTGGCAACTAATACGACTCACTATAGGGTGGATAGGCCAGGGTACTAC-3′ and 5′-ATGTGGCATCATTTAGGTGACACTATAGAAGACCAATTGCAAAATAAAATAAAAATCC-3′ was used as a template. One microgram (1 µg) of each transcript was immobilized to Hybond-N^+^ membrane (GE Healthcare). The membranes were prehybridized in a hybridization mix [37] for 24 h at 55 °C. Then, the membranes were hybridized with IVRA products for 16 h at 55 °C, washed with 5× SSPE (0.9 M NaCl, 50 mM NaH_2_PO_4_, 5 mM EDTA, pH 7.4) and 0.05% SDS, and exposed to Phosphor Imager screens. To test the specificity of the capture probes, the membranes were also hybridized with ^32^P-labeled minus and plus transcripts of the SFV4-HA and Tmed described above. The labeled transcripts were prepared using a T7 or SP6 Transcription Kit according to the manufacturer’s instructions, except for that a nucleotide mix containing 20 µM ATP, UTP, CTP, and GTP and 0.12 µM (10 µCi) of α-^32^P-CTP (Perkin Elmer) were used.

### 2.7. ^35^S-Labeling and Protein Analyses

Protein concentrations were measured by the Coomassie blue method [40] using BSA as a standard. The ^35^S-l-methionine and ^35^S-l-cysteine labeling was performed using PNS prepared from mock BSR cells or cells co-transfected with P123^HA^4 and Tmed. The ^35^S-labeling was performed in the same buffer conditions as IVRA by incubating the samples with 22 µCi of EasyTag™ EXPRESS^35^S Protein Labeling Mix (Perkin Elmer) for 1 h at 30 °C. Unicorporated label was removed using RNase-free Micro Bio-Spin™ P-30 Gel Columns (Bio-rad), and proteins were separated by SDS-polyacrylamide gel electrophoresis (SDS-PAGE) with 4% and 12% (*w*/*v*) acrylamide concentrations in the stacking and separation gels, respectively. SDS-polyacrylamide gels were stained with Coomassie blue to show total protein composition. All ^35^S-labeled proteins were detected by drying the gels and exposing them to Phosphor Imager screens. If samples were heat inactivated, they were incubated for 5 min at 96 °C before the labeling. If translation inhibitors were used, samples were incubated with puromycin (100 µg/mL; GE Healthcare) or cycloheximide (100 µg/mL; Sigma-Aldrich) for 60 min at 4 °C before the labeling. The same pre-incubation was also used when translation inhibitors were tested in IVRA.

For Western blotting, proteins were separated by SDS-PAGE with 4% and 10% (*w*/*v*) acrylamide concentrations in the stacking and separation gels, respectively, and transferred to Amersham^TM^ Protan^TM^ Nitrocellulose Blotting Membrane (GE Healthcare). After blocking with 5% (*w*/*v*) non-fat dry milk powder in Tris-buffered saline (TBS), membranes were incubated with rabbit polyclonal antibodies against SFV nsP1, nsP2, nsP3, or nsP4 [41] or mouse monoclonal anti-HA antibody (Sigma-Aldrich) in TBS with 5% milk and 0.1% (*v*/*v*) Tween-20. Mouse monoclonal antibody against β-actin (Sigma-Aldrich) was used as a cytosolic marker. Secondary antibodies IRDye^®^ 800CW donkey anti-rabbit IgG (LI-COR Biosciences, Lincoln, NE, USA) and Alexa Fluor 680 donkey anti-mouse IgG (Invitrogen) were used for fluorescent detection of the primary antibodies with an Odyssey system (LI-COR). Quantification was performed using Image Studio Software (LI-COR).

## 3. Results

### 3.1. Isolation of Active Replication Complexes from SFV-Infected Cells

**Figure 1:**
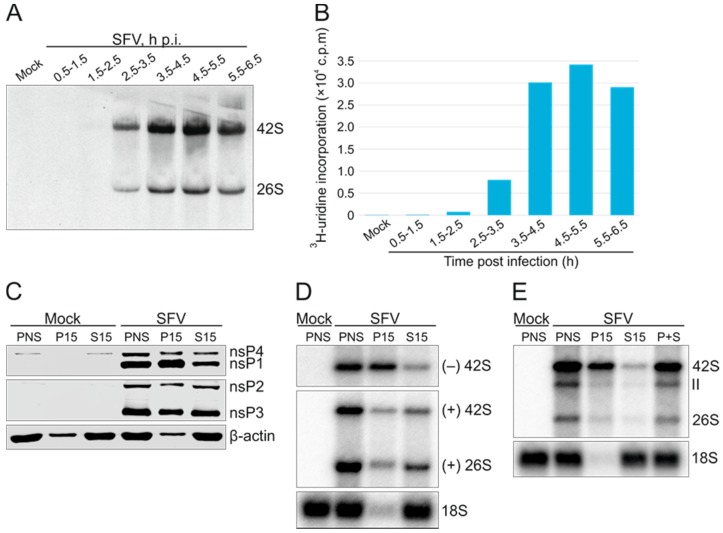
Kinetics of Semliki Forest virus (SFV) RNA synthesis and isolation of active replication complexes for in vitro analysis. (**A**) SFV-infected baby hamster kidney (BHK) cells were pulse-labeled with ^3^H-uridine for 1 h during the time periods indicated. After labeling, RNA was isolated, analysed by denaturing agarose gel electrophoresis, and visualized by fluorography. The positions of genomic and subgenomic RNA are indicated; (**B**) Liquid scintillation counts of the same RNA as in A; (**C**) Expression of the replicase proteins and their distribution between the P15 and S15 fractions as studied by Western blotting with specific antibodies. Distribution of the cytosolic marker β-actin is also shown. Post-nuclear supernatant (PNS) was prepared at 4 h post infection (p.i.), and equivalent amounts of PNS, P15, and S15 were analysed from mock- and SFV-infected cells. Data are a representative example of two independent experiments; (**D**) Distribution of SFV minus- and plus-strand RNA determined by in-gel hybridization with a probe specific to minus- or plus-strand RNA. The probe against plus-strand RNA detects both genomic and subgenomic RNA. As a control, a probe against ribosomal 18S RNA was used; (**E**) RNA synthesizing activity of various subcellular fractions analysed in vitro by determining incorporation of ^32^P-CTP into RNA. After an in vitro replication assay (IVRA), RNA was analysed in a denaturing agarose gel. P+S indicates a mixture of equal volumes of P15 and S15 from SFV-infected cells. 18S rRNA was detected by in-gel hybridization of the same gel. Representative experiments are shown in (**D**) and (**E**).

Metabolic labeling of SFV RNA synthesis with ^3^H-uridine was used to determine the optimal time point for the isolation of active SFV RCs. BHK cells were infected with an MOI of 50, and the incorporation of label into viral genomic (42S) and subgenomic (26S) RNA species became detectable after 1.5 h.p.i. (Figure 1A,B). The rate of ^3^H-uridine incorporation into viral RNA reached a maximum level at 4–5 h.p.i. Thus, SFV-infected cells were harvested at 4 h.p.i. and homogenized in isotonic buffer to prepare PNS. Typically, the PNS samples had a total protein concentration of ~3 mg/mL. To further isolate the RCs, PNS was separated into a P15 pellet and S15 supernatant by differential centrifugation at 15,000× *g*, and the distribution of viral nsPs as well as RNA was studied. The cytosolic marker β-actin and 18S rRNA were predominantly found in the S15 fractions (Figure 1C,D). Western blotting showed that all four nsPs were present in PNS, as well as in the P15 and S15 fractions (Figure 1C). The P15 fraction contained the majority of nsP1 (~90%) and about half of nsP4. Approximately 30% and 40% of nsP2 and nsP3 were found in P15, respectively. In-gel hybridizations showed that the P15 fraction contained the majority (~80%) of the minus-strand RNA present in the PNS, while a minor fraction of the genomic plus-strand RNA (~20%) was found in P15 (Figure 1D).

The RNA-synthesizing activity present in the PNS, P15, and S15 fractions was analysed by measuring the incorporation of ^32^P-CTP into viral RNA in an IVRA (Figure 1E). No labeled RNA was detected in the mock PNS, while the PNS prepared from SFV-infected cells synthesized three RNA species corresponding to SFV genomic and subgenomic RNA, as well as an RNA species designated as RNA II, in line with other alphaviruses [25,33]. The genomic RNA was the major product synthesized in vitro, and RNA II was only detected in the ^32^P-labeled reaction products of the in vitro assay, not in the ^3^H-uridine-labeled viral RNA synthesized in cells (Figure 1A,E). This might be due to the short half-life of RNA II in the infected cells or due to the lack of sensitivity in detecting ^3^H-labeled RNA compared to ^32^P-labeled molecules. Approximately 9% and 49% of the in vitro replication activity present in the PNS was recovered in the S15 and P15 fractions, respectively (Figure 1E). The membrane fraction prepared from severe acute respiratory syndrome (SARS)-coronavirus-infected cells is inactive in replication in vitro unless a cytosolic fraction is added in the reaction mixture [42]. However, mixing of the S15 and P15 did not restore the full replication activity of SFV (69% of that in the PNS), indicating that the factors in the S15 fraction are dispensable for replication and that the differential centrifugation likely caused damage to RCs, leading to a partial loss of activity. The slight boost after mixing P15 and S15 was most likely due to the cellular NTP pool present in S15.

### 3.2. Application of the In Vitro Replication Assay to the Trans-Replication System

BSR cells were co-transfected with the SFV replicase and template plasmids to determine if the RCs isolated from the trans-replication system are active in RNA synthesis in vitro. PNS was prepared from the transfected cells at 16 h post transfection using the same method as optimized for SFV-infected cells. The wild-type replicase polyprotein, P1234, as well as the polymerase inactive mutant, P1234^GAA^, were included in this study. In addition, two partially uncleaved polyproteins were used. In P1^2^34, cleavage sites between nsP1 and nsP2 as well as nsP2 and nsP3 have been mutated, resulting in the polyprotein P123 and nsP4. In P12^CA^3, the active site of the nsP2 protease has been mutated, and nsP4 is expressed from a separate plasmid as an ubiquitin–nsP4 fusion. The Tmed plasmid that expresses a 2963-nt RNA containing the 5′ and 3′ untranslated regions (UTRs) and the subgenomic promoter was provided as a template plasmid [26].

**Figure 2:**
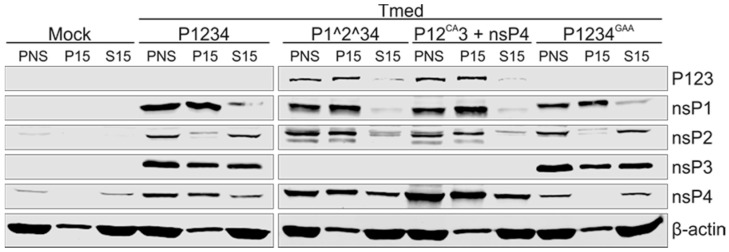
Distribution of nsPs expressed in transfected cells. BSR cells were transfected with the replicase plasmid(s) indicated at the top and Tmed template plasmid. At 16 h post transfection, PNS was prepared and fractionated into P15 and S15, and nsPs were detected by Western blotting with specific antibodies. The polyprotein P123 was recognized using anti-nsP3 antibody. β-actin was used as a cytosolic marker. Equivalent amounts of PNS, P15, and S15 were loaded. Two independent experiments were performed and one representative result is shown here.

**Figure 3:**
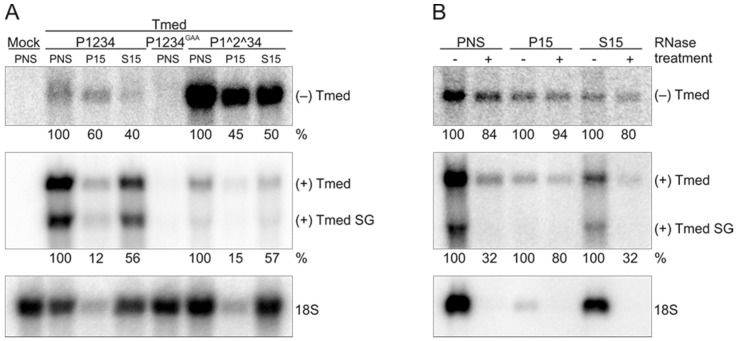
Distribution and stability of endogenous minus- and plus-strand RNA in P15 and S15 fractions prepared from transfected BSR cells. (**A**) PNS was prepared at 16 h post transfection, and total RNA was isolated from equivalent amounts of PNS, P15, and S15 followed by in-gel hybridization with probes that detect either minus- or plus-strand RNA. Subgenomic (SG) RNA is indicated by Tmed SG. Numbers below the lanes indicate the percentage of ^32^P-label detected in the genomic RNA compared to PNS. For the plus-strand RNA, the genomic Tmed was quantified. As a control, a probe against ribosomal 18S RNA was used; (**B**) PNS, P15, and S15 from cells co-transfected with the P1^2^34 and Tmed constructs were treated with RNase A/T1 under low salt conditions, and RNA was detected as in A. Numbers below the lanes indicate the percentage of ^32^P-signal in genomic RNA compared to the untreated sample. Representative experiments are shown.

Similar to the PNS from SFV-infected cells, PNS samples prepared from transfected cells gave a total protein concentration of approximately 3 mg/mL and all four nsPs were detected (Figure 2). The results of the fractionation of transfected-cell PNS into S15 and P15 were also comparable to those obtained with virus-infected cells, as β-actin and 18S rRNA were mostly found in the S15 fractions (Figure 2 and Figure 3). nsP4 was enriched in P15 (~50–70%), except in P1234^GAA^, where the majority of nsP4 was found in S15 (~80%). The P15 fraction of the cleavable constructs, P1234 and P1234^GAA^, contained the majority of nsP1 (~90 and 80%, respectively). Approximately 30% and 20% of nsP2 and ~50 and 40% of nsP3 were found in P15 of P1234 and P1234^GAA^, respectively. The polyprotein P123 was detected when cells were transfected with the uncleavable constructs, P1^2^34 or P12^CA^3, and the majority was distributed in P15 (~80 and 90%, respectively). Besides the polyprotein P123, bands recognized by anti-nsP1 and anti-nsP2 antibodies were detected in these samples. Their size was slightly smaller compared to nsP1 and nsP2 in the cleavable constructs, and thus they most likely represent unspecific cleavage products. Approximately 90% of the band detected by the anti-nsP1 antibody was found in P15. However, the unspecific cleavage product of nsP2 was also mainly found in the membrane fraction (~80%) in contrast to nsP2 in cells infected with SFV or transfected with the wild-type replicase, in which the majority of nsP2 was recovered in the cytosolic fraction (Figure 1 and Figure 2). No processed nsP3 was observed in cells transfected with the uncleavable constructs, which may be due to its instability and rapid turn-over. The only major difference observed between cells transfected with the two uncleavable constructs was that the P12^CA^3 + nsP4-transfected cells contained more nsP4, most likely due to its expression from a separate plasmid.

We next assessed the presence and distribution of endogenous RNA in the transfected samples. No signal was detected when RNA from mock-transfected or P1234^GAA^ + Tmed-transfected cells was analysed by in-gel hybridizations with probes specific for the template RNA (Figure 3A). Both minus- and plus-strand RNA were detected in PNS when the wild-type replicase and uncleavable constructs together with the template were expressed (Figure 3A). Cells expressing the uncleavable construct P1^2^34 + Tmed contained a considerably higher amount of the minus-strand RNA compared to those expressing the wild-type construct P1234 + Tmed. Cells transfected with the wild-type replicase and Tmed contained significantly higher amounts of the genomic and subgenomic plus strands when compared to the uncleavable replicase (Figure 3A). In both cases, the S15 fraction contained more plus-strand RNA, while the minus-strand RNA was distributed more evenly. Thus, it was studied if the minus-strand RNA is in a nuclease-protected form in both the membrane and cytosolic fractions. PNS, P15, and S15 fractions from P1^2^34 + Tmed were treated with RNase A/T1 under low salt conditions to digest both single- and double-stranded RNA. The digestion of 18S rRNA was observed in all treated samples, confirming that the RNase treatment was effective (Figure 3B). More minus-strand RNA was digested in PNS and S15 than in P15, but all fractions contained at least 80% of the minus-strand RNA present in their corresponding untreated sample (Figure 3B). This indicates that the minus-strand RNA was mostly protected from the nucleases and not in a freely accessible form. Furthermore, most of the plus-strand RNA present in the PNS and S15 was digested, while in P15 the plus-strand RNA was protected. This confirms the idea that the plus-strands in replicative intermediates within the RC are membrane-protected, while they are released from the RCs into the cytosol in a more nuclease-sensitive form (Figure 3B).

**Figure 4:**
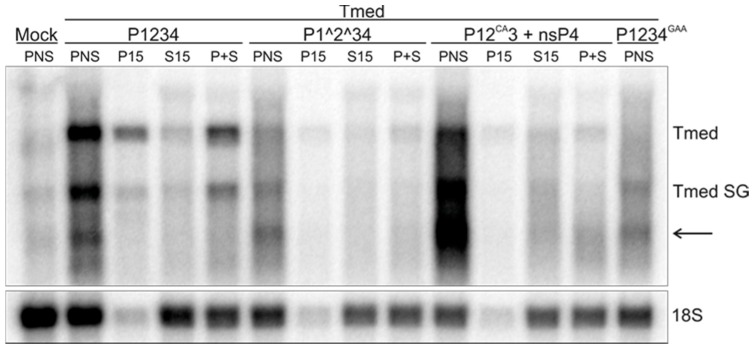
In vitro activity of replication complexes formed by expressing different SFV replicase constructs and a template in the trans-replication system. BSR cells were transfected and PNS, P15, and S15 were prepared as in Figure 3. Replication activity was determined by measuring the incorporation of ^32^P-CTP into viral RNA after separation in denaturing agarose gels. The genomic and subgenomic RNA are indicated by Tmed and Tmed SG, respectively. P+S indicates a reaction containing equal volumes of P15 and S15 fractions, and each reaction contained an equivalent amount of PNS, P15, or S15. The arrow indicates an unspecific band. Ribosomal 18S RNA was detected by in-gel hybridization of the same gel. Data are representative of at least two independent experiments.

To study replication activity, IVRAs were performed with the samples prepared from the transfected cells. PNS from both mock-transfected cells and those transfected with the inactive polymerase mutant (P1234^GAA^ + Tmed) gave some background, most likely from the T7 polymerase that is expressed in BSR cells (Figure 4). However, the wild-type replicase with the template was highly active in synthesizing both genomic and subgenomic RNA. RNA II was not observed, which may be due to its short half-life in combination with a lower activity of the RCs from transfected cells compared to those from infected cells. The P15 and S15 fractions prepared from cells transfected with P1234 + Tmed contained 38% and 15% of the replication activity present in the PNS, respectively (Figure 4). A combination of P15 and S15 gave a yield of 53%. In contrast to the wild-type replicase, the uncleavable constructs P1^2^34 and P12^CA^3 + nsP4 replicated the endogenous template poorly, although they produced RNA species with sizes corresponding to both genomic and subgenomic Tmed. However, the smaller RNA species was detected in the polymerase mutant as well and thus might be the result of another (background) activity. In addition, an unspecific product was observed in all constructs with a size below that of the subgenomic RNA. The higher expression levels of nsP4 most likely explain why P12^CA^3 + nsP4 produced more labeled RNA than P1^2^34 (Figure 2 and Figure 4).

### 3.3. Polarity of SFV RNA Synthesized In Vitro

**Figure 5:**
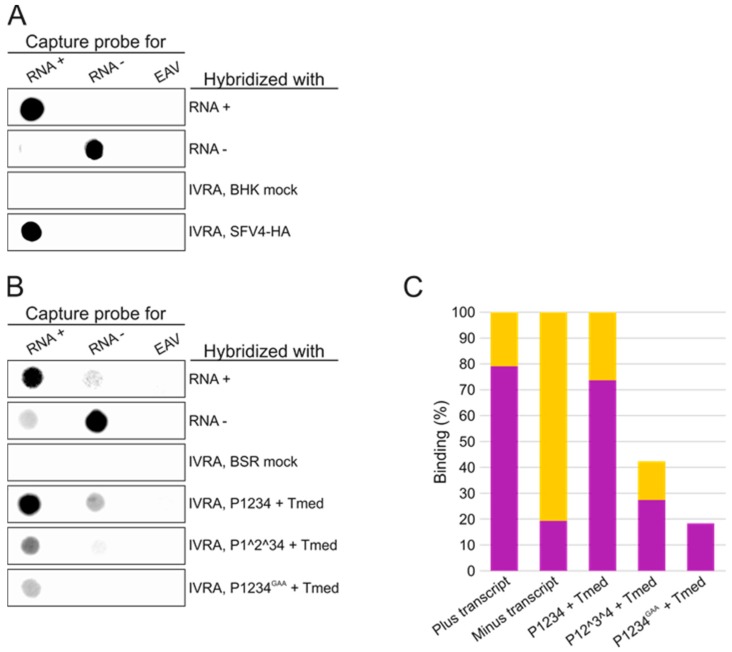
Determination of the polarity of in vitro synthesized RNA. (**A**) RNA probes with the sequence of the 3′ end of the SFV genome, recognizing minus-strand RNA (RNA−), or its complementary sequence, recognizing plus-strand RNA (RNA+), were immobilized on membranes. As a negative control, an unrelated transcript (equine arteritis virus (EAV)) was also immobilized. The membranes were then hybridized with ^32^P-labeled SFV transcripts of positive or negative polarity as well as ^32^P-labeled IVRA products. IVRA reactions with PNS samples from mock and SFV-infected cells were included; (**B**) RNA probes with the plus sequence of Tmed, recognizing minus-strand RNA (RNA−), or its complementary sequence, recognizing plus-strand RNA (RNA+), were immobilized on membranes as well as EAV transcript followed by hybridization with ^32^P-labeled Tmed plus and minus transcripts as well as ^32^P-labeled IVRA products. PNS samples from both mock and transfected cells were included; (**C**) binding of Tmed plus and minus transcripts as well as IVRA products to the capture probes was quantified from two independent experiments. The binding of the IVRA products made by replication complexes (RCs) from P1^2^34 or P1234^GAA^ + Tmed-transfected cells were normalized to the binding of the IVRA products made by RCs from P1234 + Tmed-transfected cells. Magenta indicates binding to the capture probe for the plus-strand RNA and orange to the capture probe for the minus-strand RNA.

We next examined the polarity of the RNA synthesized in vitro by the RCs isolated from SFV-infected BHK and transfected BSR cells. These RNA species were compared to in vitro transcripts of SFV or Tmed of positive or negative polarity. Some ^32^P-labeled IVRA products were denatured and hybridized with membrane-immobilized capture probes specific to positive or negative polarity SFV RNA. A capture probe corresponding to 1–2042 nt of EAV genome was used as a (negative) control for binding specificity. The IVRA products synthesized by SFV RCs strongly hybridized with the capture probe for the plus-strand RNA, indicating that SFV RCs mainly produced RNA of positive polarity in vitro (Figure 5A). Capture probes specific to the Tmed template of the trans-replication system gave more background, and thus the signal was quantified (Figure 5B,C). The IVRA products made by RCs from P1234 + Tmed-transfected cells showed similar binding to the capture probes as the plus-strand RNA transcript of Tmed. The RNA products synthesized by RCs formed upon expression of P1^2^34 + Tmed also produced a similar binding pattern as the plus-strand RNA transcript. The IVRA products made by P1234^GAA^ + Tmed showed significant binding only to the plus-RNA capture probe and the binding was approximately 67% of the binding to the plus-RNA capture probe detected with P1^2^34 + Tmed, suggesting that the signal generated by this uncleavable replicase was mostly or in part due to some background activity.

### 3.4. Effect of Ongoing Translation on In Vitro RNA Synthesizing Activity

**Figure 6:**
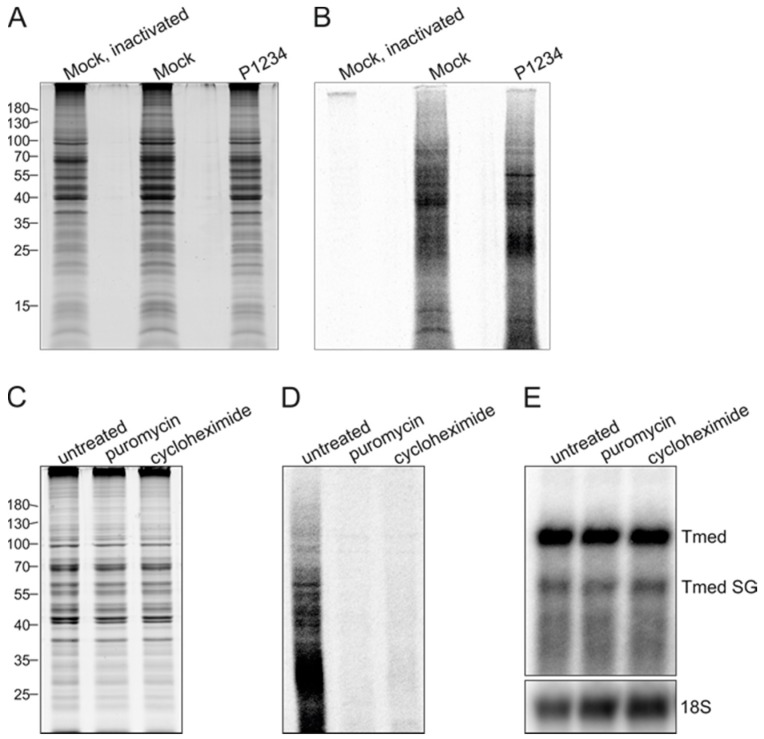
In vitro RNA-synthesizing activity is not dependent on translation. BSR cells were either mock-transfected or co-transfected with the P1234 and Tmed constructs, and PNS was prepared at 16 h post transfection. (**A**) Protein profiles of PNS samples in an SDS-polyacrylamide gel stained with Coomassie blue. One of the mock PNS samples was heat inactivated, and then all samples were incubated with ^35^S-l-methionine and ^35^S-l-cysteine. Numbers on the left indicate the molecular masses (kDa) of marker proteins; (**B**) a Phosphor Imager screen was exposed to the same gel as in A to detect ^35^S-labeled proteins; (**C**) PNS samples from the cells transfected with P1234 + Tmed were treated with translation inhibitors, puromycin or cycloheximide, and then incubated with ^35^S-methionine and ^35^S-l-cysteine. Protein profiles in an SDS-polyacrylamide gel stained with Coomassie blue are shown; (**D**) A Phosphor Imager screen was exposed to the same gel as in C to detect ^35^S-labeled proteins; (**E**) P1234 + Tmed PNS was treated with translation inhibitors as in C followed by an IVRA. 18S rRNA was detected by in-gel hybridization of the same gel. The results of one of two independent experiments is shown.

To determine if in vitro RNA synthesizing activity requires ongoing protein synthesis (which might result in the de novo formation of complexes) we studied the effect of translation inhibitors. We first established whether translation occurred when PNS samples were incubated under IVRA conditions by metabolic labeling of protein synthesis with ^35^S-Met and ^35^S-Cys. Metabolic labeling was performed on mock PNS, heat-inactivated mock PNS, and PNS prepared from BSR cells co-transfected with the wild-type replicase and Tmed template. Coomassie staining demonstrated that these samples contained similar amounts of total protein (Figure 6A). A synthesis of ^35^S-labeled proteins was readily detected in mock PNS and replicase-expressing PNS (Figure 6B), despite the fact that the IVRA reaction conditions might not be optimal for protein synthesis. Heat inactivation of PNS prior to metabolic labeling abolished the incorporation of ^35^S (Figure 6B), confirming that the radioactive signal in lanes 2 and 3 of Figure 6B is the result of translation. The translation inhibitors puromycin and cycloheximide were added to PNS prior to metabolic labeling, and this inhibited the de novo production of proteins in vitro as the incorporation of ^35^S into proteins was no longer observed, while the total protein content of the samples was practically unaffected (Figure 6C,D). When these translation inhibitors were added during IVRA, the RNA synthesizing activity remained unchanged (Figure 6E), demonstrating that ongoing protein synthesis is not required for RNA synthesis in vitro.

### 3.5. Purification of Replicase Protein-Containing Membranes and Addition of Exogenous Template RNA

**Figure 7:**
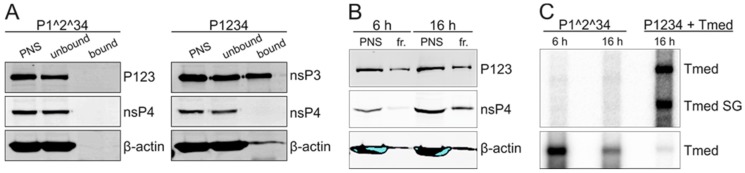
Exogenous template RNA is not replicated in vitro. (**A**) BSR cells were transfected with the polyprotein construct P1^2^34 or P1234 and cells were collected at 16 h post transfection followed by homogenization and HA-affinity capture. The HA tag was in nsP3. After PNS was incubated with HA-specific agarose beads, they were washed and boiled with Laemmli sample buffer. The presence of polyprotein P123, nsP3, nsP4, and β-actin in PNS as well as in unbound and bound fractions was studied by Western blotting. P123 and nsP3 were detected using an antibody against the HA tag; (**B**) BSR cells were transfected with the polyprotein construct P1^2^34, cells were collected at 6 or 16 h post transfection, and membranes were purified from PNS using flotation centrifugation. A Western blot analysis shows the presence of P123, nsP4, and β-actin in the PNS and membrane fractions (fr.); (**C**) IVRA was performed with the flotation fractions by adding 1 µg of Tmed in vitro transcript. As a control, replication activity from the endogenous template is shown for PNS prepared from BSR cells co-transfected with the P1234 and Tmed constructs. The lower panel shows the presence of exogenous Tmed transcript in the membrane fractions after the IVRA, detected by in-gel hybridization from the same gel using a probe specific to the plus-stranded Tmed.

To gain more insight into the function of the early RC formed by the polyprotein P123 and polymerase nsP4, we expressed the uncleavable construct P1^2^34 without the template RNA in BSR cells. Cells were harvested at 6 and 16 h post transfection, and membranes containing the polyprotein and polymerase were mixed with an in vitro transcribed RNA to study if they were able to replicate an exogenous template. However, the added RNA was rapidly degraded in the P15 fraction, and thus RC-containing membranes were further purified from PNS. First, we tested HA-specific affinity capture using HA-tagged nsP3. Western blotting showed that the polyprotein P123 or nsP4 did not bind to the HA-specific agarose beads (Figure 7A). When the cleavable polyprotein P1234 was expressed in cells, we observed binding of nsP3 (Figure 7A). However, nsP4 did not bind in this case either, indicating that bound nsP3 was not in a complex with the other nsPs. Thus, we next tested the purification of membranes using flotation centrifugation in an iodixanol step-gradient. The light-scattering band was collected and Western blotting confirmed the presence of the polyprotein P123 and nsP4 in the membrane fractions (Figure 7B). Next, the membrane fractions were provided with an exogenous template, the positive polarity in vitro transcript of Tmed, and IVRAs were performed. In-gel hybridization confirmed the presence of significant amounts of exogenous Tmed RNA transcript in both membrane fractions after the IVRA had been performed (Figure 7C, lower panel). The membrane fraction prepared from the cells harvested at 6 h post transfection contained more in vitro transcript than the sample prepared 16 h post transfection, suggesting that RNase activity was somewhat higher in the flotation samples prepared from cells at the later time point post transfection. Although exogenous Tmed transcript was present throughout the incubation with the membrane fractions, no incorporation of ^32^P-CTP into viral RNA was detected (Figure 7C, upper panel), despite the fact that template RNA was still present at the end of the reaction (Figure 7C, lower panel).

Besides the T7 promoter-driven replicase constructs that were expressed in BSR T7/5 cells, we also used BHK-21 cells that were transfected with plasmids that express replicase proteins under control of the cytomegalovirus promoter. P1^2^34 replicase-containing membrane fractions from these cells were also unable to replicate the exogenous template. To exclude that the lack of activity was due to the template itself, we also tested a smaller RNA template, Tshort (1529 nt), without success. To give the complex more time to form, we also pre-incubated P1^2^34 replicase-containing membrane preparations with an exogenous template for 1 h at 4 °C before the reaction was performed, and we tested different reaction times from 30 min to 2 h. None of these experimental variations resulted in the replication of the exogenous template.

## 4. Discussion

**Figure 8:**
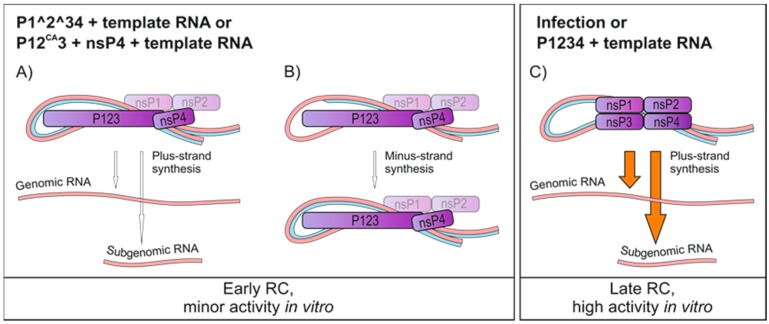
Schematic representation of the possible activities of various RCs that can be produced with the trans-replication system. (**A**,**B**) When cells are transfected with the partially uncleavable replicase and template constructs, PNS shows very little activity in vitro indicated by the white arrows. A minor amount of plus-strand RNA is made, and it is also possible that RCs finish initiated minus strands. These complexes represent early RCs; (**C**) when cells are infected or transfected with the wild-type replicase and template constructs, the prepared PNS is highly active in vitro and synthesizes RNA of positive polarity, both genomic and subgenomic, indicated by the orange arrows. These complexes are late RCs.

Herein, we analysed SFV replication using a combination of in vitro activity and trans-replication systems. The results described in this study revealed the main requirements for RNA synthesis in vitro and are summarized in Figure 8. At least three independently prepared batches of PNS, P15, and S15 from infected and transfected cells produced very similar results (activities), showing the robustness of the system.

Highly active RNA synthesis was observed in vitro only when RCs were isolated from cells containing the fully processed SFV replicase, either resulting from virus infection or transfection with the wild-type replicase and template plasmids (Figure 1 and Figure 4). Highly diluted PNS from infected cells was routinely used in the IVRA, demonstrating the robustness of the in vitro RNA-synthesizing activity of SFV. Albeit less active, the RCs isolated from transfected cells were also functional in making both genomic and subgenomic RNA (Figure 4). A combination of the in vitro RNA synthesis assay and trans-replication system can also be applied to study the replication of CHIKV, as a trans-replication system is available for this highly pathogenic alphavirus as well [28].

Infection and the trans-replication system only yielded isolated RCs that synthesized exclusively plus-strand RNA (Figure 5). These complexes thus represent late-stage RCs formed by nsP1 to nsP4 and are dedicated to the synthesis of plus strands [8,21]. Here, the optimal time point to isolate active RCs from SFV-infected cells was around 4 h.p.i. (Figure 1), and thus the observed plus-strand synthesis is consistent with SFV minus-strand synthesis ceasing between 3 and 4 h.p.i. in mammalian cells [43]. Furthermore, our and previous metabolic labeling studies of SFV-infected cells also indicate that viral replication is maximal at this time [32]. Endogenous minus strands and replication activity were mainly found in the membrane fraction prepared from SFV-infected cells, while endogenous plus strands were more abundant in the cytosolic fraction (Figure 1). The same has been observed with CHIKV-infected cells [25], and confirms the idea that the membranous spherules accommodate the minus-strand RNA as well as the active replication complex [23,24,25]. Interestingly, the cytosolic fractions from the trans-replication system contained more minus-strand RNA than those from infected cells. However, the minus-strand RNA in the cytosolic fraction from transfected cells was insensitive to RNases (Figure 3), suggesting it is in spherules or other protective structures. This is also supported by the observation that the ratio of the activity between the S15 and P15 fractions was higher in the samples prepared from the transfected cells than in those obtained from infected cells (Figure 1 and Figure 4). These differences in spherule distribution might be explained by their subcellular location. With the infection conditions described here, SFV spherules are mainly located in small endocytic vesicles and perinuclear cytopathic vacuoles [18]. In the trans-replication system, spherules mainly stay at the plasma membrane [29].

The partially uncleaved replicase protein produced considerably higher amounts of the minus-strand RNA and considerably smaller amounts of the plus-strand RNA in cells, compared to the wild-type replicase (Figure 4). It has been observed that the early RCs consisting of P123 and nsP4, although primarily synthesizing minus strands, are also able to make plus strands [6,8,21,24], albeit inefficiently compared to late-stage RCs. However, it is intriguing how dramatic the difference in the minus-strand RNA synthesis was. If we assume that minus strands represent RCs, our results imply that the partially uncleaved replicase is able to make significantly higher numbers of RCs compared to the wild-type replicase. This suggestion is in line with the requirement of partially uncleaved replicase to form spherules without the template [29]. Furthermore, Western blotting showed that in all replicase constructs the polymerase nsP4 was enriched in the membrane fraction, with the exception that in P1234^GAA^ it did not become membrane-associated but mainly remained free in the cytosol (Figure 2). Because the spherule assembly without active viral replication is achieved only if P23 remains uncleaved [29], RCs are not formed using a fully cleaved construct P1234^GAA^, and consequently nsP4 stays cytosolic, explaining our results. Thus, the recruitment of nsP4 to the RC requires its catalytic activity and the presence of a template RNA.

Besides replication, active translation was observed in the SFV in vitro system (Figure 6), similar to PNS prepared from SARS-coronavirus-infected cells, for which ongoing protein synthesis was not required for in vitro replication activity [42]. Likewise, the activity of isolated SFV RCs did not require ongoing translation in the in vitro assay (Figure 6), suggesting that (the bulk of) the activity that is measured in our assay can be attributed to the existing isolated RCs and that new RCs that might be formed (if at all) de novo during the reaction do not contribute significantly. Translation inhibitors did not boost replication either, indicating that ribosomes caused no significant stall of the polymerase complex. In SFV-infected cells, which synthesize both minus and plus strands, translation inhibitors cause selective shut off of the minus-strand RNA synthesis [43]. However, overproduction of the nonstructural proteins does not lead to increased RNA synthesis in SFV-infected cells [44].

The in vitro replication and capture probe experiments showed that the partially uncleaved replicases had a very poor in vitro replication activity on an endogenous template (Figure 4 and Figure 5). The amount of radiolabeled RNA that was produced in vitro by P1^2^34 was only slightly above the background levels that were observed when the inactive polymerase mutant was used, although P1^2^34 produced high levels of the minus-strand RNA in cells (Figure 3, Figure 4 and Figure 5). This indicates that after P123 and nsP4 have formed early RCs and synthesized the minus-strand in cells, they do not continue to make additional minus strands (in vitro). This is supported by the observation that a translation inhibitor blocks continuous minus-strand synthesis in SFV- and SINV-infected cells [43,45]. Furthermore, membranes containing the polyprotein P123 and polymerase nsP4 were unable to initiate RNA synthesis in vitro using an exogenous template (Figure 7). As it has been shown that the partially uncleaved replicase forms spherules without the template [29], we speculate that the proteins were sequestered in spherules and did not have access to and so could not recruit the cytosolic template.

Introduction of the HA-affinity tag in the nonstructural protein 4B (NS4B) has been used to purify hepatitis C virus (HCV) RCs [38]. NS4B is a membrane protein, and the scaffolding protein HCV RCs and affinity capture resulted in an enrichment of DMVs capable of the de novo synthesis of HCV RNA. Thus, we also probed a similar approach to isolate SFV replicase proteins by tagging nsP3, which is known to tolerate insertions [18]. However, HA-affinity capture could not be used to purify complexes of P123 and nsP4 or complexes of nsP3 and nsP4; only nsP3 bound to the capture beads (Figure 7) and most likely represented cytoplasmic aggregates of nsP3 [46,47], supporting our hypothesis that nsPs engaged in replication are sequestered in spherules. Consequently, the results indicate that the minus-strand synthesis requires that RCs or spherules are assembled at the same time as the minus-strand synthesis is initiated, and this was not achieved in vitro because the replicase proteins had already formed spherules. The recent cryo-electron tomographic reconstruction of Flock House virus (FHV) spherules also suggests that the minus-strand is synthesized simultaneously with spherule formation [23]. However, although the minus-strand synthesis seems to depend on the RC assembly, SFV spherule formation does not depend on viral RNA replication [29].

It is intriguing that the uncleavable polyprotein P123 and polymerase nsP4 of SINV are able to start the minus-strand synthesis from an exogenous template in a recombinant vaccinia virus expression system [30] in contrast to the results obtained with our SFV expression system. However, SINV P123 and nsP4 are also able to form spherules without the template [29], and it will be interesting to test if these replicase proteins are able to replicate an exogenous RNA template, when expressed analogously to the SFV system we have used in this study. Furthermore, it will be important to compare this system to the vaccinia virus-based expression system used in the previous SINV studies. Interestingly, SINV nsP4 purified from *Escherichia coli* synthesizes RNA in vitro if combined with a P123-membrane fraction and template RNA [16], and thus it might be that in the vaccinia virus-based expression system there are P123 and nsP4 proteins which have not yet formed spherules and are thus able to recruit and replicate an exogenous template.

In this study, we showed that SFV polyprotein P123 and polymerase nsP4 were active in RNA replication only in cells, and after isolation these early RCs were unable to effectively synthesize RNA in vitro. In contrast, the polymerase nsP4 together with nsP1, nsP2, and nsP3 was highly active in vitro and these late RCs could be isolated for in vitro analyses. The next step to shed light on the mechanisms and molecular details that underlie the differences between the early and late RCs would be to analyse their formation from template recruitment to RNA synthesis and membrane deformation. This will, however, require that we freeze RC assembly at different stages. It has been shown that P123 alone does not form spherule structures [29]. Thus, an interesting model system could be provided by the purified nsP4 combined with a P123-membrane fraction and template RNA [16]. For SINV, such a system is active in de novo RNA synthesis, indicating that RC assembly is achievable in vitro if the template RNA is provided before nsP4 has associated with the polyprotein P123.

## References

[1] King A.M.Q., Adams M.J., Carstens E.B., Lefkowitz E.J. (2012). Virus Taxonomy: Ninth Report of the International Commitee on Taxonmy of Viruses.

[2] Koonin E.V., Dolja V.V., Krupovic M. (2015). Origins and evolution of viruses of eukaryotes: The ultimate modularity. Virology.

[3] Amraoui F., Failloux A.B. (2016). Chikungunya: An unexpected emergence in Europe. Curr. Opin. Virol..

[4] Tsetsarkin K.A., Chen R., Weaver S.C. (2016). Interspecies transmission and chikungunya virus emergence. Curr. Opin. Virol..

[5] Strauss J.H., Strauss E.G. (1994). The alphaviruses: Gene expression, replication, and evolution. Microbiol. Rev..

[6] Lemm J.A., Rice C.M. (1993). Roles of nonstructural polyproteins and cleavage products in regulating Sindbis virus RNA replication and transcription. J. Virol..

[7] Lemm J.A., Rice C.M. (1993). Assembly of functional Sindbis virus RNA replication complexes: Requirement for coexpression of P123 and P34. J. Virol..

[8] Lemm J.A., Rumenapf T., Strauss E.G., Strauss J.H., Rice C.M. (1994). Polypeptide requirements for assembly of functional Sindbis virus replication complexes: A model for the temporal regulation of minus- and plus-strand RNA synthesis. EMBO J..

[9] Ahola T., Kääriäinen L. (1995). Reaction in alphavirus mRNA capping: Formation of a covalent complex of nonstructural protein nsP1 with 7-methyl-GMP. Proc. Natl. Acad. Sci. USA.

[10] Spuul P., Salonen A., Merits A., Jokitalo E., Kaariainen L., Ahola T. (2007). Role of the amphipathic peptide of Semliki forest virus replicase protein nsP1 in membrane association and virus replication. J. Virol..

[11] Das P.K., Merits A., Lulla A. (2014). Functional cross-talk between distant domains of chikungunya virus non-structural protein 2 is decisive for its RNA-modulating activity. J. Biol. Chem..

[12] Hardy W.R., Strauss J.H. (1989). Processing the nonstructural polyproteins of sindbis virus: Nonstructural proteinase is in the C-terminal half of nsP2 and functions both in cis and in trans. J. Virol..

[13] Vasiljeva L., Merits A., Golubtsov A., Sizemskaja V., Kaariainen L., Ahola T. (2003). Regulation of the sequential processing of Semliki Forest virus replicase polyprotein. J. Biol. Chem..

[14] Kim D.Y., Reynaud J.M., Rasalouskaya A., Akhrymuk I., Mobley J.A., Frolov I., Frolova E.I. (2016). New world and old world alphaviruses have evolved to exploit different components of stress granules, FXR and G3BP proteins, for assembly of viral replication complexes. PLoS Pathog..

[15] Li C., Debing Y., Jankevicius G., Neyts J., Ahel I., Coutard B., Canard B. (2016). Viral macro domains reverse protein ADP-ribosylation. J. Virol..

[16] Rubach J.K., Wasik B.R., Rupp J.C., Kuhn R.J., Hardy R.W., Smith J.L. (2009). Characterization of purified Sindbis virus nsP4 RNA-dependent RNA polymerase activity in vitro. Virology.

[17] Paul D., Bartenschlager R. (2013). Architecture and biogenesis of plus-strand RNA virus replication factories. World J. Virol..

[18] Spuul P., Balistreri G., Kääriäinen L., Ahola T. (2010). Phosphatidylinositol 3-kinase-, actin-, and microtubule-dependent transport of Semliki Forest virus replication complexes from the plasma membrane to modified lysosomes. J. Virol..

[19] Frolova E.I., Gorchakov R., Pereboeva L., Atasheva S., Frolov I. (2010). Functional Sindbis virus replicative complexes are formed at the plasma membrane. J. Virol..

[20] Thaa B., Biasiotto R., Eng K., Neuvonen M., Gotte B., Rheinemann L., Mutso M., Utt A., Varghese F., Balistreri G. (2015). Differential phosphatidylinositol-3-kinase-Akt-mTOR activation by Semliki Forest and Chikungunya viruses is dependent on nsP3 and connected to replication complex internalization. J. Virol..

[21] Shirako Y., Strauss J.H. (1994). Regulation of Sindbis virus RNA replication: Uncleaved P123 and nsP4 function in minus-strand RNA synthesis, whereas cleaved products from P123 are required for efficient plus-strand RNA synthesis. J. Virol..

[22] Kallio K., Hellström K., Balistreri G., Spuul P., Jokitalo E., Ahola T. (2013). Template RNA length determines the size of replication complex spherules for Semliki Forest virus. J. Virol..

[23] Ertel K.J., Benefield D., Castano-Diez D., Pennington J.G., Horswill M., den Boon J.A., Otegui M.S., Ahlquist P. (2017). Cryo-electron tomography reveals novel features of a viral RNA replication compartment. Elife.

[24] Kallio K., Hellström K., Jokitalo E., Ahola T. (2015). RNA replication and membrane modification require the same functions of alphavirus nonstructural proteins. J. Virol..

[25] Albulescu I.C., Tas A., Scholte F.E., Snijder E.J., van Hemert M.J. (2014). An in vitro assay to study chikungunya virus RNA synthesis and the mode of action of inhibitors. J. Gen. Virol..

[26] Spuul P., Balistreri G., Hellström K., Golubtsov A.V., Jokitalo E., Ahola T. (2011). Assembly of alphavirus replication complexes from RNA and protein components in a novel trans-replication system in mammalian cells. J. Virol..

[27] Hellström K., Kallio K., Meriläinen H.M., Jokitalo E., Ahola T. (2016). Ability of minus strands and modified plus strands to act as templates in Semliki Forest virus RNA replication. J. Gen. Virol..

[28] Utt A., Quirin T., Saul S., Hellström K., Ahola T., Merits A. (2016). Versatile trans-replication systems for Chikungunya virus allow functional analysis and tagging of every replicase protein. PLoS ONE.

[29] Hellström K., Kallio K., Utt A., Quirin T., Jokitalo E., Merits A., Ahola T. (2017). Partially uncleaved alphavirus replicase forms spherule structures in the presence and absence of RNA template. J. Virol..

[30] Lemm J.A., Bergqvist A., Read C.M., Rice C.M. (1998). Template-dependent initiation of Sindbis virus RNA replication in vitro. J. Virol..

[31] Barton D.J., Sawicki S.G., Sawicki D.L. (1991). Solubilization and immunoprecipitation of alphavirus replication complexes. J. Virol..

[32] Clewley J.P., Kennedy S.I. (1976). Purification and polypeptide composition of Semliki Forest virus RNA polymerase. J. Gen. Virol..

[33] Wielgosz M.M., Huang H.V. (1997). A novel viral RNA species in Sindbis virus-infected cells. J. Virol..

[34] Buchholz U.J., Finke S., Conzelmann K.K. (1999). Generation of bovine respiratory syncytial virus (BRSV) from cDNA: BRSV NS2 is not essential for virus replication in tissue culture, and the human RSV leader region acts as a functional BRSV genome promoter. J. Virol..

[35] Ulper L., Sarand I., Rausalu K., Merits A. (2008). Construction, properties, and potential application of infectious plasmids containing Semliki Forest virus full-length cDNA with an inserted intron. J. Virol. Methods.

[36] Keränen S., Kääriäinen L. (1974). Isolation and basic characterization of temperature-sensitive mutants from Semliki Forest virus. Acta. Pathol. Microbiol. Scand. B Microbiol. Immunol..

[37] Scholte F.E., Tas A., Martina B.E., Cordioli P., Narayanan K., Makino S., Snijder E.J., van Hemert M.J. (2013). Characterization of synthetic Chikungunya viruses based on the consensus sequence of recent E1–226V isolates. PLoS ONE.

[38] Paul D., Hoppe S., Saher G., Krijnse-Locker J., Bartenschlager R. (2013). Morphological and biochemical characterization of the membranous hepatitis C virus replication compartment. J. Virol..

[39] Van Dinten L.C., den Boon J.A., Wassenaar A.L., Spaan W.J., Snijder E.J. (1997). An infectious arterivirus cDNA clone: Identification of a replicase point mutation that abolishes discontinuous mRNA transcription. Proc. Natl. Acad. Sci. USA.

[40] Bradford M.M. (1976). A rapid and sensitive method for the quantitation of microgram quantities of protein utilizing the principle of protein-dye binding. Anal. Biochem..

[41] Kujala P., Ikäheimonen A., Ehsani N., Vihinen H., Auvinen P., Kääriäinen L. (2001). Biogenesis of the Semliki Forest virus RNA replication complex. J. Virol..

[42] Van Hemert M.J., van den Worm S.H., Knoops K., Mommaas A.M., Gorbalenya A.E., Snijder E.J. (2008). SARS-coronavirus replication/transcription complexes are membrane-protected and need a host factor for activity in vitro. PLoS Pathog..

[43] Sawicki D.L., Sawicki S.G. (1980). Short-lived minus-strand polymerase for Semliki Forest virus. J. Virol..

[44] Sawicki S.G., Sawicki D.L. (1986). The effect of overproduction of nonstructural proteins on alphavirus plus-strand and minus-strand RNA synthesis. Virology.

[45] Gorchakov R., Frolova E., Sawicki S., Atasheva S., Sawicki D., Frolov I. (2008). A new role for ns polyprotein cleavage in Sindbis virus replication. J. Virol..

[46] Frolova E., Gorchakov R., Garmashova N., Atasheva S., Vergara L.A., Frolov I. (2006). Formation of nsP3-specific protein complexes during Sindbis virus replication. J. Virol..

[47] Gorchakov R., Garmashova N., Frolova E., Frolov I. (2008). Different types of nsP3-containing protein complexes in Sindbis virus-infected cells. J. Virol..

